# EGNOS 1046 Maritime Service Assessment

**DOI:** 10.3390/s20010276

**Published:** 2020-01-03

**Authors:** Deimos Ibáñez Segura, Adrià Rovira Garcia, María Teresa Alonso, Jaume Sanz, José Miguel Juan, Guillermo González Casado, Manuel López Martínez

**Affiliations:** 1 Research Group of Astronomy and Geomatics (gAGE), Universitat Politècnica de Catalunya (UPC), 08034 Barcelona, Spain; adria.rovira@upc.edu (A.R.G.); maria.teresa.alonso@upc.edu (M.T.A.); jaume.sanz@upc.edu (J.S.); jose.miguel.juan@upc.edu (J.M.J.); guillermo.gonzalez@upc.edu (G.G.C.); 2 European GNSS Agency (GSA), 170 00 Prague, Czech Republic; manuel.lopezmartinez@gsa.europa.eu

**Keywords:** Global Navigation Satellite System (GNSS), Satellite Based Augmentation System (SBAS), International Maritime Organization (IMO)

## Abstract

The present contribution evaluates how the European Geostationary Navigation Overlay System (EGNOS) meets the International Maritime Organization (IMO) requirements established in its Resolution A.1046 for navigation in harbor entrances, harbor approaches, and coastal waters: 99.8% of signal availability, 99.8% of service availability, 99.97% of service continuity and 10 m of horizontal accuracy. The data campaign comprises two years of data, from 1 May 2016 to 30 April 2018 (i.e., 730 days), involving 108 permanent stations located within 20 km of the coast or in islands across the EGNOS coverage area, EGNOS corrections, and cleansed GPS broadcast navigation data files. We used the GNSS Laboratory Tool Suite (gLAB) to compute the reference coordinates of the stations, the EGNOS solution, as well as the EGNOS service maps. Our results show a signal availability of 99.999%, a horizontal accuracy of 0.91 m at the 95th percentile, and the regions where the IMO requirements on service availability and service continuity are met. In light of the results presented in the paper, the authors suggest the revision of the assumptions made in the EGNOS Maritime Service against those made in EGNOS for civil aviation; in particular, the use of the EGNOS Message Type 10.

## 1. Introduction

The International Maritime Organization (IMO) is the United Nations (UN) authority for the safety, security, and environmental performance of international shipping, recalled in the Safety of Life at Sea (SOLAS) Convention of 1974 [1]. The Convention was updated and amended through Resolutions by the IMO Assembly, the IMO Maritime Safety Committee (MSC), or other IMO bodies. In Resolution A.815 (revoked) of 1995, then A.953 (revoked) of 2003, and finally A.1046 of 2011 (in force) [2,3], the IMO Assembly provided the procedure and requirements for recognizing suitable radio navigation systems as components of the World-Wide Radio Navigation System (WWRNS). WWRNS represents an international qualification of the system to allow its use for safety of navigation in merchant shipping.

The Global Positioning System (GPS) and GLONASS were recognized as a part of the WWRNS in 1996 [4], whereas Beidou and Galileo were recognized in 2014 and 2016, respectively [5,6]. IMO MSC requires in its Resolution MSC.401 [7] of 2015 the use of at least two independent Global Navigation Satellite Systems (GNSS) constellations in order to compute a reliable Position Velocity Time (PVT) estimate. Regarding the necessary level of resilience and integrity in the PVT solution, MSC.401 recommends the shipborne equipment to have the facility to process augmentation data to improve the performance of space based PVT signals in terms of [8,9,10,11]:

- Accuracy: the difference between the estimated and actual position.
- Availability: the percentage of time that the services of the system are usable under stated conditions.
- Continuity: the capability of the system to perform its function under stated conditions without scheduled and/or unscheduled interruptions during the intended operation.
- Time To Alert (TTA): the maximum allowable time elapsed from the onset of the navigation system being out of tolerance until the equipment enunciates the alert.
- Integrity: a measure of the trust that can be placed in the correctness of the information supplied by the system, including the ability of a system to provide timely and valid warnings to the user when the system should not be used for navigation.

Three types of Augmentation Systems (AS) are available, depending on how the external additional information is broadcast: satellite based, ground based, or receiver autonomous. Altogether, AS support applications in sectors such as the aviation, maritime, railways, and road [11,12,13,14,15]. Our study focuses on the Satellite Based Augmentation Systems (SBAS).

SBAS augments core GNSS constellations providing ranging, integrity, and correction information by means of:

- A network of ground reference stations that monitor the GNSS Signal in Space (SiS).
- Master stations that collect and process reference station data and generate SBAS messages.
- Uplink stations to send the SBAS messages to Geosynchronous Equatorial Orbit (GEO) satellites.
- Transponders on these GEO satellites that broadcast the different SBAS Messages Types (MTs).

**Figure 1:**
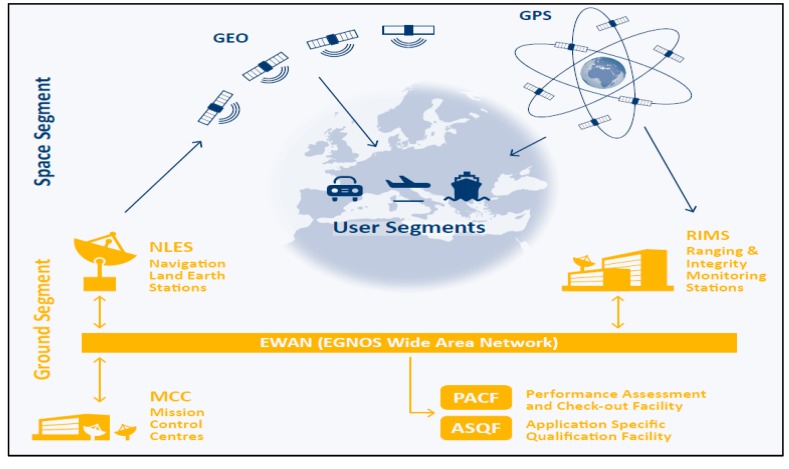
European Geostationary Navigation Overlay System (EGNOS) architecture. GPS data are collected by the Ranging Integrity Monitoring Stations (RIMSs) and sent to the Mission Control Centre (MCC), which generates the EGNOS corrections and integrity data. This information is sent in a message to the Navigation Land Earth Stations (NLES) to be uplinked to the GEO satellites, which broadcast the EGNOS message to the users. This figure is from [17], available at www.gsa.europa.eu.

Figure 1 depicts the architecture of the European Geostationary Navigation Overlay Service (EGNOS), the European SBAS. From the different SBAS available, the present study selected EGNOS, which has provided vertical and lateral guidance to civil aircraft since 2011 [16].

In order to cover a wide area like a continent, SBAS treats errors affecting GNSS SiS taking into account the nature of the error [18,19]. Thus, SBAS corrections are computed separately and distributed to the users using different MT; see Table A1 in the Appendix A.

The SBAS corrections are organized into two categories: clock-ephemeris corrections and ionospheric corrections. This information, even for individual satellites, is distributed across several individual messages, MT0 to MT28, which are coordinated through Issues Of Data (IOD).

Fast Corrections (FCs) are scalar values common to all SBAS users, primarily removing clock errors. In contrast, Long Term Corrections (LTCs) are given as a vector and affect users in a different way at different locations. LTCs primarily remove ephemeris errors and also account for the “slow varying” clock trend. FC and LTC have associated confidence intervals (i.e., sigmas) to weight the satellite data properly in the navigation filter, when SBAS users compute both the PVT solution and its confidence bounds (i.e., the protection levels).

FCs are broadcasted in MT2 to MT5, while MT25 is devoted to long term corrections. MT24 is a mixed message where both FC and LTC are broadcasted. MT25 allows saving bandwidth in case there are few satellites remaining to broadcast FC and LTC at a given time. Finally, the delay at the upper layer of the atmosphere (i.e., the ionosphere) is corrected with MT26.

Real-time applications entail delays and message losses. This is accounted for in MT7, which provides FCs degradation parameters to add uncertainty to the estimated range corrections, as well as time-outs to avoid using the FC going beyond its validity period. Finally, MT10 provides degradation factors mainly for the LTCs and ionosphere.

In the context of the maritime domain, the International Association of Lighthouse Authorities (IALA) published in 2017 guidelines [20] on the retransmission of SBAS corrections via marine radio beacons and the Automatic Identification System (AIS).

The aim of the present study is the evaluation of EGNOS for maritime navigation compliance with the last version of the IMO requirements for the WWRNS, which dates from 2011, namely the IMO Assembly Resolution A.1046 [3] and expressed in Table 1. In what follows, we refer to EGNOS 1046 Maritime Service in accordance to the numbering of the IMO Resolution. An analysis of the requirements was done in [21] and hereafter.

The position update rate of 2 s is met by design when using GNSS. In terms of horizontal accuracy, the unaugmented Standard Positioning Service Performance Standard (SPS PS) already commits to 9 m 95% horizontal error in an average user location; see Table 3.8-3 in [22]. Once augmented by EGNOS, the European Satellite Services Provider (ESSP) reports [23] horizontal accuracies better than 0.8 m at the 95% level.

In terms of SiS availability, ESSP reported in [23] 100% of SiS availability adding the two GEO satellites broadcasting EGNOS corrections. The TTA of 10 s is also achieved by the design of current SBAS, which assures a TTA down to 6 s [24]. The main concern of the authors and the great interest of the present study is whether the EGNOS 1046 Maritime Service can meet the requirement of 99.97% for discontinuity of the service using a Continuity Time Interval (CTI) of 15 min. It is noted that the requirement posed by the International Civil Aviation Organization (ICAO) is 99.95% [24], but using a CTI of 15 s.

The paper is organized as follows. The next section describes the data retrieved from public servers that were used for the study. Then, the methodology and the different GNSS processing steps are described in detail. The results show how the EGNOS 1046 Maritime Service meets the IMO requirements, including a discussion after looking at the results. Finally, the main research conclusions are highlighted and summarized.

## 2. Dataset

**Figure 2:**
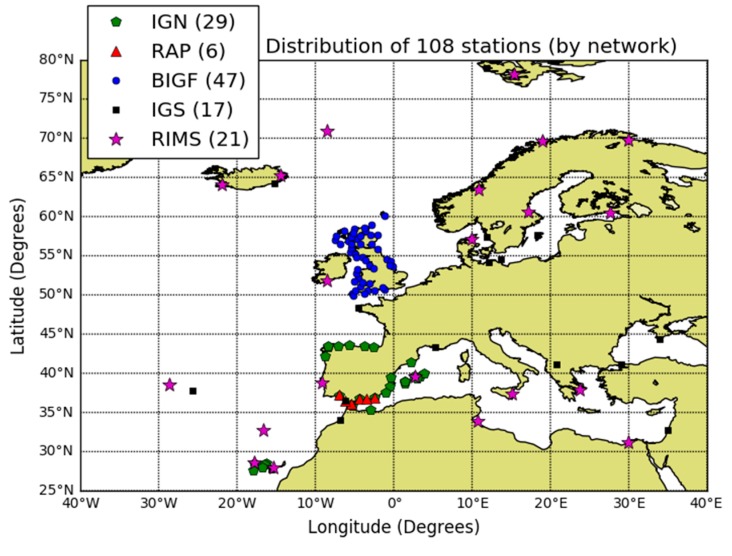
Distribution of permanent stations used for the study: 6 stations of the Andalusian Positioning Network (RAP); 29 stations of the Spanish National Geographic Institute (IGN); 35 stations from the British Isles continuous GNSS Facility (BIGF); 17 stations from the International GNSS Service (IGS); and 21 EGNOS Ranging Integrity Monitoring Stations (RIMSs). Specifically, at each RIMS, we used the receiver designated as “A” from the two co-located receivers “A” and “B”.

The data campaign was comprised of two years of data, from 1 May 2016 to 30 April 2018 (i.e., 730 days). For every day, we downloaded GPS broadcast navigation messages and final precise orbits and clocks from the International GNSS Service (IGS) [25], EGNOS differential corrections from the Navigation and Time Monitoring Facility (NTMF) FTP server (ftp://serenad-public.cnes.fr/SERENAD0/FROM_NTMFV2/MSG/) that belongs to National Centre for Space Studies (CNES), and observation files stored with the Receiver Independent Exchange Format (RINEX) format. RINEXs recorded GNSS code pseudoranges and carrier phase measurements at a 1 Hz sampling rate for 108 permanent stations located at a maximum of 20 km from the coast, belonging to five different networks depicted in Figure 2. In the analyzed period, three different Pseudo Random Numbers (PRN) were used to broadcast EGNOS corrections; see Table 2.

## 3. Methodology

We used our in-house open-source GNSS laboratory (gLAB) tool suite [27], from the European Space Agency (ESA) and gAGE/UPC, as the engine to evaluate the EGNOS 1046 Maritime Service; in particular, the SBAS maritime template in gLAB [28]. Before the main GNSS data processing, two preliminary computations were needed:

- Computing the reference position for each of the 108 permanent stations, by the Precise Point Positioning (PPP) technique [29,30], using the PPP template in gLAB. This PPP, in static mode over 24 hours of data, provides accuracies at the centimeter level [10], which are close to two orders of magnitude better than the expected accuracy of the SBAS positioning. Therefore, these reference positions can be used with good confidence as a ground truth to evaluate the accuracy of EGNOS across the European continent.
- Cleansing the RINEX GPS navigation files containing the satellite ephemerides, following the methodology presented in [31]. The cleansing (external to gLAB) removed potential errors and inconsistencies such as data logging errors and/or hardware/software bugs, losses of navigation messages, and different transmission times, among others. In contrast, the EGNOS files are already consolidated by CNES and did not require further processing.

### 3.1. Area Maps

The first part of the study focused on the evaluation of the availability and continuity of the EGNOS 1046 Maritime Service. This examination merely assessed the geometry of the augmented satellites, hence only requiring the cleansed navigation files and the consolidated EGNOS messages, following a fault-free receiver approach; that is a receiver that is assumed to be in continuous signal tracking, without cycle slips, and with all GPS navigation data and EGNOS corrections available.

The examined area covered the longitude range from 40° W to 50° E and the latitude range from 10° N to 80° N, using a resolution of squared pixels of 1° per 1° and a sampling rate of 1 s. We used the generic SBAS maritime template in gLAB [28] with minor modifications, such as setting the mask for the satellite elevation to 7 degrees.

Note that Table 1 does not provide a requirement value on the Horizontal Alarm Limits (HAL) and Vertical Alarm Limits (VAL), whereas the earlier IMO Resolution A.915 stated AL requirements [32]. Therefore, those values were disabled in gLAB by taking huge HAL and VAL values of 100 km. Instead, a filtering criterion based on the Position Dilution Of Position (PDOP) and Horizontal Dilution of Positioning (HDOP) was applied to ensure that the position information could be reliably used for navigation purposes. Indeed, according to [32], epochs whose satellite geometries have PDOP ≥ 6 and HDOP ≥ 4 were excluded.

#### 3.1.1. Availability

The service availability calculation was straightforward, but for completeness, it is explicated here:

*Availability* (%) = 100 × *up time/total time* (1)

where the total time is 730 days, or equivalently 63,072,000 s. Having in mind the requirement of 99.8% expressed in Table 1, (1) allowed up to 35 h and 2 min of down time for the entire data campaign.

#### 3.1.2. Continuity

The service continuity over a Continuity Time Interval (CTI) of 15 min was computed using two methodologies. The first approach evaluated the service continuity by means of a sliding window, according to the Radio Technical Commission for Aeronautics (RTCA) Minimum Operational Performance Standards (MOPS) for GPS [18]:

*Continuity sliding window* (%) = 100 × (1 − *NEAD/total epochs*) (2)

where the Number of Epochs Affected by Discontinuities (NEAD) is the total sum of epochs with an available solution prior to a discontinuity inside a CTI. When one discontinuity occurs, the previous 900 epochs (i.e., CTI of 15 min using a sampling rate of 1 s) with the solution are accumulated in NEAD. If the elapsed time from the last discontinuity is lower than 900 epochs or the CTI, only the actual number of elapsed epochs since the last discontinuity is accumulated in NEAD. The total epochs are the total number of epochs with a solution.

This first approach (2) based on the sliding window took into account only the total number of epochs with the solution and the number of epochs between each discontinuity. Therefore, the continuity result was accurate and consistent independent of the length of the period processed and the CTI. This was especially important when a large number of availability fluctuations occurred in the borders of coverage of EGNOS, as later shown in the results.

The second approach evaluated the service continuity using the formula defined by the International Association of Maritime Aids to Navigation and Lighthouse Authorities (IALA) in its guidelines for monitoring of Differential GNSS (DGNSS) services [33]:

*Continuity fixed window* (%) = 100 (1 − *CTI/MTBF*) = 100 × (1 − n *CTI/total time*) (3)

where the CTI is 15 min and the Mean Time Between Failures (MTBF) is computed as the total number of epochs processed (total time) independently if these epochs had an available solution or not, divided by the total number of discontinuities (n) occurring during the processed period.

This second approach was easier to compute than (2), as it only required determining n. However, it assumed that only one discontinuity occurred during a time span of one CTI (fixed window) (which was only valid when processing large periods of time and, most of the time, the service was available). When this assumption was not true, two downsides appeared: the first one was in the case where many transitions from available to unavailable and vice versa occurred in a short period of time, which would make (3) perform worse than (2), and in the case where there were very few available epochs, (3) could yield negative values, which makes no sense. The second downside was when, most of the time, the system was unavailable (as it occurred in the edges of the EGNOS coverage area) and a very small number of discontinuities occurred, and (3) yielded a continuity very close to one, which was, in the opinion of the authors, misleading.

It is interesting to calculate the number of discontinuities allowed by (2) and (3), before reaching the requirement of 99.97% expressed in Table 1. In (2), the result depends on the spacing between the discontinuity events. In order to seize the worst case, NEAD = 900·nmax epochs was assumed as if every event affected an entire CTI. Then, the total of the epochs was equal to 63,072,000 − 900 × *nmax*. This yielded a maximum of 21 discontinuities of a length equal to 900 s, which equaled to 5 h 15 min. In contrast, (3) yielded also 21 discontinuities, but of any duration. Hence, in the case that each discontinuity lasted 1 s (i.e., the most demanding case), the approach of (3) only allowed 21 s of unavailability in 2 years, which represents an availability of 99.99997% of the time; a very tight requirement to be met.

### 3.2. Accuracy

The second part of the study focused on evaluating the accuracy of the EGNOS 1046 Maritime Service; that is the deviation of the coordinates obtained with a single frequency EGNOS solution with respect to the reference position obtained with dual frequency PPP. In this case, besides the previous modifications to the SBAS Maritime Template, gLAB was configured as follows:

- Setting the pre-fit outlier detector threshold to 100 m, i.e., two orders of magnitude of the expected error, to filter out local errors (e.g., multipath or receiver hardware malfunctioning).
- Selecting the GEO satellite providing the lowest 3D protection level, in order to achieve the best performance. Nevertheless, the impact of this GEO selection (switching) was very small.

In this regard, for every epoch at a 1 Hz sampling rate and for every station, the Horizontal and Vertical Positioning Error (HPE and VPE) were computed together with the Horizontal and Vertical Protection Levels (HPL and VPL) according to [18]. The results were then accumulated to obtain histograms and the Cumulative Distribution Function (CDF) of the PEs and PLs together with its Root Mean Square (RMS), mode, mean, and percentiles to have a robust statistical characterization. Finally, we completed the assessment computing the Worst Integrity Ratio (WIR), which was the largest ratio between the PE and the PL.

Note that despite IMO Resolution A.1046 [3] only considers the horizontal component, and the current paper addressed the vertical component in line with the earlier IMO Resolutions A.915 [32] and MSC.233 [34]. Moreover, later IMO guidelines for shipborne Position, Navigation and Timing (PNT) data processing [35] also impose requirements on the vertical component for some port and restricted water operations. Therefore, the assessment of the VPE and VPL was considered of interest and is presented hereafter.

## 4. Results

### 4.1. SiS Availability

Table 3 reports the EGNOS SiS outages experienced by the operational GEOs broadcasting EGNOS corrections from 1 May 2016 to 30 April 2018. The reception percentage of SBAS messages from at least one of the satellites (PRN 120, 123, or 136) was 99.999%, as only 14 s of simultaneous SiS absence were recorded. Thus, the requirement on the signal availability of 99.8% was fulfilled.

### 4.2. Service Availability

**Figure 3:**
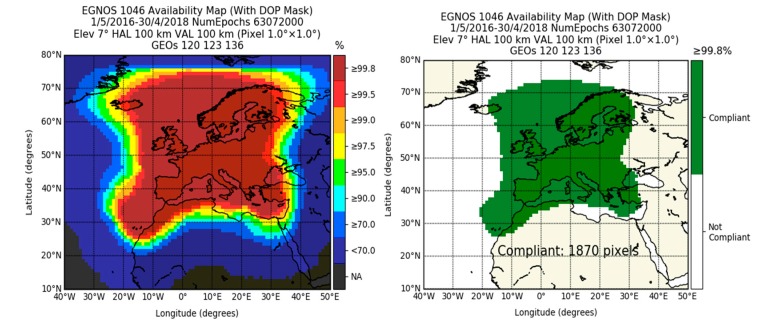
Availability of the EGNOS 1046 Maritime Service (left panel) and its compliance with the IMO requirement of 99.8% (right panel). Elev., Elevation Mask; DOP, Dilution Of Position; HAL, Horizontal Alarm Limits; VAL, Vertical Alarm Limits.

Figure 3 depicts the service availability maps computed over the whole period, from 1 May 2016 to 30 April 2018. Each point of the grid depicts the percentage of the number of epochs available using (1). In order to avoid any smoothing of the results, no interpolation was used. The panel on the left depicts the availability from 70% to 99.8%, whereas the right panel depicts in a binary representation its compliance with respect to the aforementioned IMO requirement of 99.8% in harbor entrances, approaches, and coastal waters.

In order to have a numerical evaluation of the percentage of the European region where the requirement was achieved, Figure 3 includes the compliance area measured in pixels, where each pixel represents a square of 1 × 1 degrees. Figure 3 shows that the most stringent IMO requirements in terms of availability in coastal waters were met in the majority of the continental area. In contrast, in the borders of coverage (e.g., the west of Iceland, east in Libyan waters), strong gradients of performance were observed. For such areas, the evaluation required a finer resolution, as performed in [36] over the Canary Islands.

### 4.3. Service Continuity

**Figure 4:**
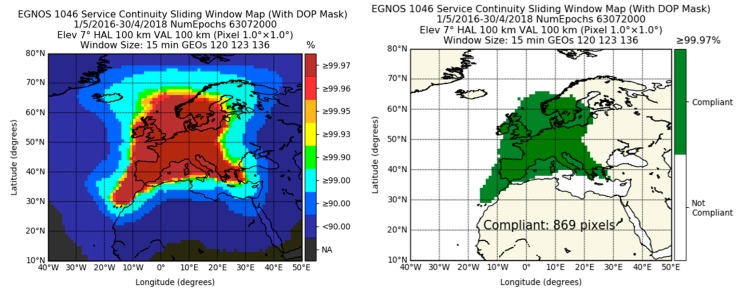

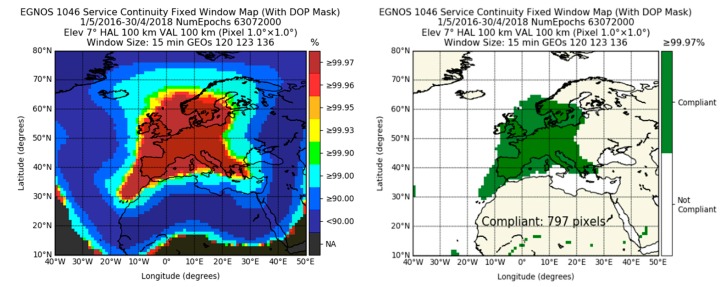
Continuity of the EGNOS 1046 Maritime Service (left column) and its compliance with the IMO requirement of 99.97% (right column). The top row uses the Radio Technical Commission for Aeronautics (RTCA) methodology (sliding window), whereas the bottom row uses the International Association of Lighthouse Authorities (IALA) methodology (fixed window), both using a window length of 15 min. The number of compliant pixels is counted within longitudes −25° W to 35° E and latitudes 25° N to 75° N.

Figure 4 depicts the service continuity computed with the methodologies of (2) and (3). That is, the top row depicts the continuity achieved using a sliding window of 15 min, whereas the bottom row shows the results computed using a fixed window of 15 min. It can be observed that the area where the continuity requirement was met with the fixed window method of (3) was 72 pixels lower than when computed with the sliding window calculation of (2). As mentioned in the Methodology Section, (3) provided unrealistic high continuity values in regions where there were only very few available epochs during the whole assessment (e.g., coordinates 40° W and 30° N or 0° E and 15° N). These unrealistic values were excluded in the computation of pixels compliant with the 99.97% requirement.

Furthermore, in areas where many transitions from available to unavailable and vice versa occurred in a short period, (3) yielded lower values of continuity than (2). This difference was especially relevant in the edge of the compliant area, i.e., around Ireland, the United Kingdom, the Baltic Sea, and the Canary Islands.

It is worth mentioning that we chose the continuity value of 99.97% for the cut. However, as suggested by the IALA Guidelines 1112 [37] and IALA recommendation R-129 [38], it is possible to relax the requirement in case the service is complemented by an IALA beacon or even to remove it as a requirement in the case of back-up (IALA Recommendation R-129, Appendix 1) for coastal navigation.

The next version of EGNOS v2.4.2 will provide new software for RIMS that will reduce the number of discontinuities, improving the continuity map, and will enlarge the service area.

### 4.4. Service Accuracy

**Figure 5:**
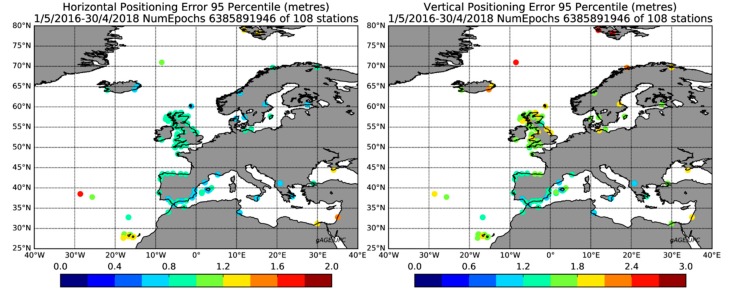

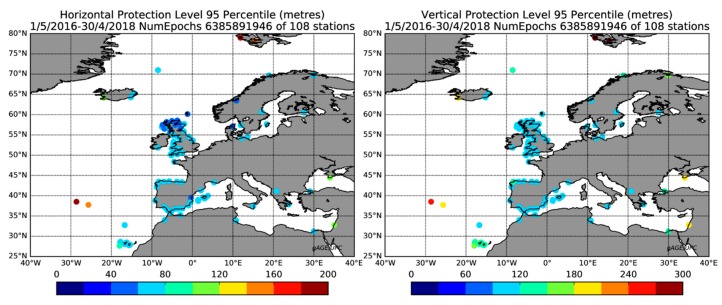
EGNOS 1046 Maritime Service 95th percentile of the positioning error (top row) and protection level (bottom row) for the horizontal (left column) and vertical (right column) when using the solution with all-in-view satellites. Color bar in meters.

Figure 5 depicts the 95th percentiles of the Positioning Errors (PEs) and Protection Levels (PLs) for the 108 stations considered in the present study. In order to represent the results in a compact manner, a circle is drawn for each position, colored according to the PE and PL values. Notice the ratio 100 in the values between the top and bottom row of the plots depicting the errors and protections, respectively. As expected, the EGNOS 1046 Maritime Service performance depended on the location of the testing station within its coverage area. In the center of the map, the PE and PL were low (cold colors), whereas the maximum values (warmest colors) were located at the edges.

In order to have a numerical evaluation, Table 4 aggregates the 6,385,891,946 epochs computed from the 108 stations depicted in Figure 2. The overall availability after aggregating all the individual stations was 93.62%. In terms of horizontal accuracy, the 95th percentile of HPE at 0.91 m fulfilled the IMO requirement of 10 m, expressed in Table 1.

**Figure 6:**
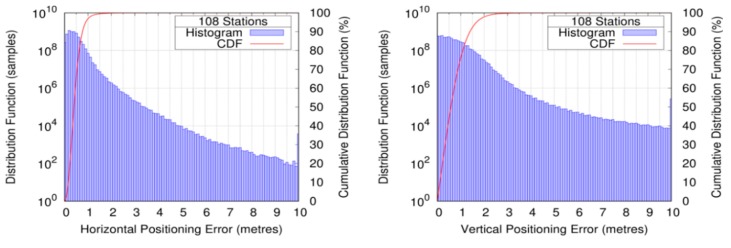

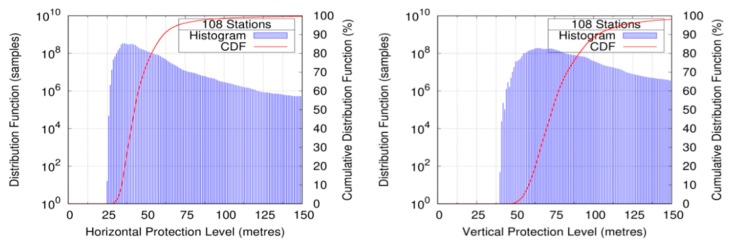
Histogram and cumulated distribution function of the positioning error (top row) and protection level (bottom row) for the horizontal (left column) and vertical (right column) components.

Figure 6 depicts the histogram (in logarithmic scale) and its corresponding Cumulative Distribution Function (CDF) for HPE, VPE, HPL, and VPL. The authors noticed that while the positioning errors were rather small, with 99.9% of 2.20 m and 2.58 m for the HPE and VPE, the corresponding protection levels started in the range of 20 m, which motivated a further analysis of the root cause, which is presented later.

### 4.5. Service Integrity

**Figure 7:**
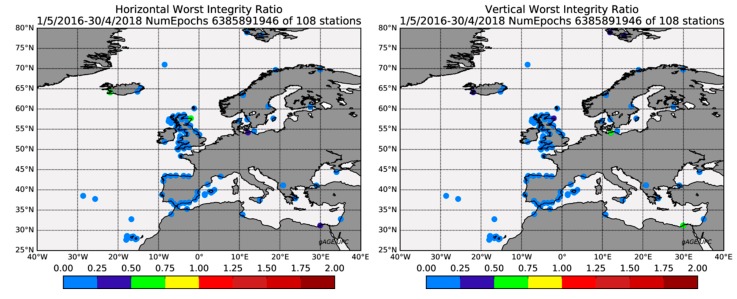
Worst integrity ratio map for the horizontal (left panel) and vertical (right panel) components. The color of the circle indicates for each station the maximum ratio between the PE and the PL for the solution with all in view satellites. Color bar in meters.

Figure 7 completes the assessment using the Worst Integrity Ratio (WIR), which is the largest ratio between the PE and its corresponding PL of the two year period. The WIR maps exhibited low values (in the bins with cold colors) for both horizontal and vertical components, evidencing the safe performance of the EGNOS 1046 Maritime Service. Indeed, the horizontal and vertical WIR were always equal to or smaller than one; thus, no Misleading Information (MI) producing PE ≥ PL occurred during the examined period.

Unlike the previous accuracy assessment, the results did not exhibit a dependency on the location of the station. Although some stations exhibited WIRs between 0.5 and 0.75, they were located near other stations with nominal WIR values at the same time. This fact evidenced that these high WIR values were probably linked to local effects (e.g., bad satellite geometries, receiver multipath) rather than to a failure of EGNOS corrections. In the latter case, all receivers within the footprint of the GEO satellite would be impacted.

## 5. Discussion

In light of the results presented in the previous section, the large values of the PL (in the range of hundreds of meters) in comparison with the ordinary PE (in the range of one meter) questioned. In this review, the assumptions made in the EGNOS 1046 Maritime Service were crosschecked with respect to those made in EGNOS for civil aviation, compliant with RTCA DO-229D [18].

**Figure 8:**
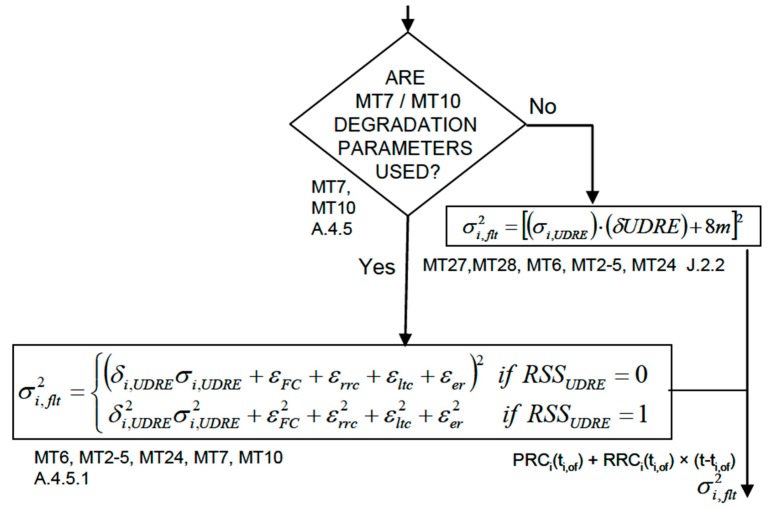
Detail of the logic of applying corrections and integrity values from SBAS (Figure S-2 from Appendix S from RTCA DO-229D [18]). This figure is copyrighted by RTCA, Inc. and used with permission. The complete RTCA document referenced may be purchased from RTCA, Inc. RTCA, Inc. 1150 18th Street NW Suite 910 Washington, DC 20036 (202) 833–9339 www.rtca.org.

The main difference that explains the discrepancy in the PL calculation of EGNOS Maritime was identified as the disabling of EGNOS Message Type 10 (MT10); see Table A1. MT10 accounted for the degradation of the fast and long term corrections with respect to time. While the civil aviation community uses the values present in MT10, the EGNOS 1046 Maritime Service does not apply them. In contrast, a conservative bound of 8 m was used, as indicated in Figure 8. This choice enlarged the PL values.

In order to evaluate the impact of applying the degradation parameters from EGNOS MT10 on the assessment presented in the previous section, the processing of the 108 stations was repeated applying MT10. Table 5 captures the difference in the positioning and the protection level domains. It can be observed that the accuracy remained practically the same (HPE and VPE rows) with an improvement of 5%, whereas the corresponding protection levels (HPL and VPL rows) were reduced by a factor of five in the 95th percentile.

## 6. Conclusions

The present contribution evaluated the availability, continuity and accuracy of the EGNOS 1046 Maritime Service for a period of 24 consecutive months. Our study confirmed the area where EGNOS was compliant with the most demanding requirements established in IMO Resolution A.1046.

We concluded that EGNOS was suitable for maritime navigation in coastal and oceanic waters. It is important to note that beyond 12 nautical miles from the coast (limit of the territorial waters), the continuity requirement was no longer needed. Therefore, the EGNOS 1046 Maritime Service area could be expanded towards the European water boundaries.

In light of the results presented in the present paper, the authors suggest using Message Type 10 when the receiver computes the coordinates using the EGNOS 1046 Maritime Service. This proposal for modification was included in the last version of the guidelines for manufacturers for the implementation of SBAS in shipborne receivers [39].

## Figures and Tables

**Table 1:** IMO requirements established in its Resolution A.1046 (27), extracted from [3].

Parameter	Navigation Phase	
	*Ocean Waters*	*Harbor Entrances, Harbor Approaches, and Coastal Waters*
Horizontal Accuracy 95%	100 m	10 m
Signal Availability	99.8%	99.8%
Service Continuity (over 15 min)	n/a	99.97%
Position update rate	2 s	2 s
Time to Alarm ^a^	Maritime safety information as soon as practicable	10 s
System Coverage	Adequate ^b^	Adequate ^b^

^a^ Generation of integrity warnings in cases of system malfunctions, non-availability, or discontinuities. ^b^ Taking into account the radio frequency environment, the coverage of the system should be adequate to provide position fixing throughout this phase of navigation.

**Table 2:** Status of GEO satellites broadcasting EGNOS corrections from 1 May 2016 to 30 April 2018 [26].

GEO Name	PRN	Orbit Slot	01 May 2016 00:00 20 March 2017 15:05	20 March 2017 15:06 21 March 2017 09:45	21 March 2017 09:46 30 April 2018 23:59
ASTRA SES-5	136	5° E	Operational	Operational	Test
INMARSAT 3F2	120	15.5° W	Operational	Operational	Operational
ASTRA-5B	123	31.5° E	Test	Operational	Operational
INMARSAT 4F2	126	64° E	Test	Test	Test

**Table 3:** Signal in Space (SiS) outages for operational GEOs broadcasting EGNOS corrections from 1 May 2016 to 30 April 2018. PRN, Pseudo Random Number.

GEO PRN	Days	SiS Gaps (s)	*Availability (%)*
120	730	64954	99.90
123	407	5323	99.98
136	323	32140	99.88
All operational GEOs	730	14	99.999

**Table 4:** Horizontal Positioning Error (HPE), Vertical Positioning Error (VPE), Horizontal Protection Level (HPL), and Vertical Protection Level (VPL) accumulating 108 stations and two years of data. For each of these metrics is depicted the availability of solution in terms of absolute and relative epochs, the percentiles (50, 68, 95, 99, 99.9), and the maximum, RMS, and mean values (in meters).

Metric	Epochs	(%)	50%	68%	95%	99%	99.9%	Max	RMS	Mean
HPE	6385891946	93.62	0.41	0.52	0.91	1.29	2.20	100.58	0.51	0.44
VPE	6385891946	93.62	0.58	0.85	1.68	2.30	3.58	2124.02	0.95	0.68
HPL	6385891946	93.62	42.52	47.77	70.32	107.48	193.58	812.66	49.00	46.40
VPL	6385891946	93.62	65.15	81.71	118.19	175.50	367.03	26,725.38	85.22	73.51

**Table 5:** Same as Table 4, but including the use of EGNOS Message Type 10 (MT10).

Metric	MT10	50%	68%	95%	99%	99.9%	Max	RMS	Mean
HPE	NO	0.41	0.52	0.91	1.29	2.20	100.58	0.51	0.44
	YES	0.39	0.49	0.86	1.20	2.07	100.39	0.48	0.41
VPE	NO	0.58	0.85	1.68	2.30	3.58	2124.02	0.95	0.68
	YES	0.56	0.81	1.57	2.15	3.47	1100.68	0.85	0.65
HPL	NO	42.52	47.77	70.32	107.48	193.58	812.66	49.00	46.40
	YES	8.60	9.46	14.98	28.21	74.38	764.16	11.11	9.66
VPL	NO	65.15	81.71	118.19	175.50	367.03	26,725.38	85.22	73.51
	YES	14.02	15.63	24.76	44.23	131.14	16,856.30	20.21	15.85

## Appendix A

**Table A1:** Summary of SBAS messages types, extracted from [18].

Id	Message Content	Message Description
0	Do Not Use for Safety Applications	MT0 alerts user about a problem in the GEO that has sent this message. After receiving MT0, all information received from this GEO (except for the almanac) must be removed, waiting 60 s until the GEO can be reused.
1	PRN Mask Assignments	MT1 contains 210 slots of 1 bit, designating the satellite PRN and the GNSS constellation (e.g., GPS uses Slots 1 to 37). Bit is 1 for monitored PRNs, with the same sequence in which the corrections are sent in other MTs.
2, 3, 4, 5	Fast Corrections	MTs provide for every 13 satellites in the PRN mask: MT2 (Sat 1 to Sat 13), MT3 (14 to 26), MT4 (27 to 39), MT5 (40 to 51), the Fast Corrections (FCs) and User Differential Range Error Indicator (UDREI).
6	Integrity Message	MT6 transmits UDREIs for up to the 51 monitored satellites to re-validate or time-out the Pseudorange Corrections (PRCs) broadcasts in MT2-MT5.
7	FC Degradation Factor	MT7 contains the time-outs to be applied to each of the 51 monitored PRNs and the system latency value, for computing the FC degradation.
9	GEO Navigation Message	MT9 contains the navigation message for one SBAS GEO usable in the navigation solution if its User Range Accuracy (URA) index is less than 15.
10	Degradation Parameters	MT10 contains the parameters to account for the degradation with respect to the time of Long Term Corrections (LTCs), FCs, Range Rate Corrections (RRCs), and ionosphere for En-Route (ER) through the Non-Precision Approach (NPA).
12	Network Time	MT12 contains time parameters to synchronize the SBAS receiver with Universal Time Coordinated (UTC).
17	GEO Almanac	MT17 contains the almanac for the GEO transmitting SBAS signals.
18	Ionospheric Mask	MT18 contains the mask of monitored Ionospheric Grid Points (IGPs), divided into bands (9 vertical and 2 horizontal), and provides IGP order.
24	Fast and Long Term Corrections	MT24 broadcasts both FC and LTC if the last FC message contains less than 7 PRNs. The first half includes FC for 6 or less satellites, whereas the second half holds the same LTC as each half of MT25.
25	Long Term Corrections (LTCs)	MT25 contains corrections to satellite coordinates and clock offsets computed from the broadcast navigation message.
26	Ionospheric Delay	MT26 contains the ionospheric delay estimations and its confidence interval at each monitored IGP, according to the mask in MT18.
27	Service Message	MT27 contains data for computing δUDRE at a (squared or triangular) region within the degradation of FC and LTC, in conjunction with MT10.
28	Clock Ephemeris Covariance Matrix	MT28 contains the satellite covariance matrix for computing δUDRE. SBAS service providers can only send either MT27 or MT28, but not both.
